# CircAMOTL1 Promotes Tumorigenesis Through miR-526b/SIK2 Axis in Cervical Cancer

**DOI:** 10.3389/fcell.2020.568190

**Published:** 2020-12-03

**Authors:** Zhengwei Sun, Sanqiang Niu, Fuxia Xu, Weidong Zhao, Rong Ma, Mingwei Chen

**Affiliations:** ^1^Department of Obstetrics & Gynecology, Anhui No. 2 Provincial People’s Hospital, Hefei, China; ^2^Department of Obstetrics & Gynecology, Bozhou People’s Hospital, Bozhou, China; ^3^Department of Obstetrics & Gynecology, The First Affiliated Hospital of USTC, Hefei, China; ^4^Department of Obstetrics & Gynecology, Anhui Women and Child Health Care Hospital, Hefei, China; ^5^Department of Urology, The First Affiliated Hospital of Soochow University, Suzhou, China

**Keywords:** circAMOTL1, miR-526b, ceRNA, SIK2, AKT, cervical carcinoma

## Abstract

**Background:**

Cervical cancer is one of the most common malignancies in women, leading to major health problems for its high morbidity and mortality. Numerous studies have demonstrated that circular RNAs (circRNAs) could be participated in the progression of multifarious diseases, especially plentiful carcinomas. CircAMOTL1 (angiomotin-like1, ID: hsa_circ_0004214), which is located on human chromosome 11:9 4532555-94533477, is involved in the occurrence of breast cancer, etc. However, the intrinsic and concrete molecular mechanism of circAMOTL1 in cervical carcinomas remained thoroughly unclear, which was also the bottleneck of circRNAs studies in cancer.

**Methods:**

The relative expression levels of circAMOTL1 and miR-526b in cervical carcinoma patients’ specimens and cervical carcinoma cell lines were detected by RT-qPCR. Through experiments including loss-function and overexpression, the biological effects of circAMOTL1 and miR-526b on the proliferation, migration, apoptosis, and tumorigenicity were explored in cervical carcinomas. Dual luciferase reporter gene analysis, western blot, and other methods were adopted to explore the circAMOTL1 potential mechanism in cervical carcinomas.

**Results:**

In our experiments, our researches displayed that circAMOTL1 was significantly higher expression in cervical carcinomas specimens and cell lines. Further experiments illustrated that the knockdown of circAMOTL1 could restrain the malignant phenotype, AKT signaling, and epithelial–mesenchymal transition (EMT) of in cervical carcinomas cells. Meanwhile miR-526b was downregulated in cervical carcinomas and even miR-526b could partially reverse circAMOTL1 function in malignant cervical tumor cells. CircAMOTL1 acts as a microRNA (miRNA) sponge that actively regulates the expression of salt-inducible kinase 2 (SIK2) to sponge miR-526b and subsequently increases malignant phenotypes of cervical carcinomas cells. In a word, circAMOTL1 acts a carcinogenic role and miR-526b serves as the opposite function of antioncogene in the cervical carcinoma pathogenesis.

**Conclusion:**

CircAMOTL1-miR-526b-SIK2 axis referred to the malignant progression and development of cervical carcinomas. CircAMOTL1 expression was inversely correlated with miR-526b and positively correlated with SIK2 mRNA in cervical cancer tissues. Thus, circAMOTL1 exerted an oncogenic role in cervical cancer progression through sponging miR-526b. Taken together, our study revealed that circAMOTL1 acted as an oncogene and probably was a potential therapeutic target for the cervical cancer.

## Introduction

Cervical cancer is one of the most common malignancies in women (Cisse et al., 2019; Feng et al., 2019; Vliet-Gregg et al., 2019; Wang et al., 2020b), leading to major health problems for high morbidity and mortality (Li et al., 2019; Ni et al., 2020; Wang et al., 2020d; Wu et al., 2020a). Cervical cancer is mainly caused by the human papilloma virus infection (Ding et al., 2019; Young et al., 2019; Cerasuolo et al., 2020), and is one of the most common gynecological malignancies, and its incidence rate ranks second among female malignancies in developing countries, and is behind breast cancer (Cisse et al., 2019; Li et al., 2019; Ni et al., 2020; Wang et al., 2020d; Wu et al., 2020a), and its mortality rate ranks third that is behind breast cancer and lung cancer. At present, surgery, radiotherapy, and chemotherapy have a better effect on early cervical cancer (Hu et al., 2019; Harkenrider et al., 2020; Rotman et al., 2020; Wang et al., 2020a), which its 5-year survival rates could reach 91.5%, but its efficacy on advanced and metastatic cervical cancers is very limited with its 5-year survival rate is only 17.3% (Wang et al., 2019; Chen et al., 2020; Du et al., 2020; Zhang et al., 2020b), meanwhile, the survival rate of recurrent cervical cancer is lower (Jayamohan et al., 2019; Wei et al., 2019; Yuan et al., 2019; Zhang et al., 2019). Therefore, the mechanism of the development and progression in cervical carcinomas is very important for early diagnosis and effective treatment of cervical cancers. How to effectively treat advanced and recurrent cervical cancers is the focus of current researches.

Recent studies have uncovered that non-coding RNA such as lncRNAs and circular RNAs (circRNAs) could play more crucial roles in various biological processes (Chen et al., 2016; Li et al., 2016a; Tran et al., 2020),including cell proliferation, migration, apoptosis, and drug resistance (Zhuang et al., 2015; Li et al., 2016b; Chen et al., 2017). CircRNAs belong to a special class of non-coding RNA molecules, which are also the latest research hotspots in the field of RNA. Different from linear RNA(with 5′ and 3′ ends), and circRNA molecule has a closed circular structure, that is not affected by RNA exonuclide, and its expression is more stable and not easy to degrade (Dong et al., 2020; Jin et al., 2020; Zhang et al., 2020a). In terms of functions, recent studies reveal that circRNA molecules are rich in microRNA (miRNA) binding sites, and act as a miRNA sponge in cells, thereby removing the inhibition of miRNA on its target genes and increasing the expression level of target genes (Di Agostino et al., 2020; Li and Wang, 2020; Liu et al., 2020). This mechanism of action is called competitive endogenous RNA (ceRNA) mechanism. By interacting with miRNAs associated with diseases, circRNAs play important regulatory roles in diseases (Dong et al., 2020; Jin et al., 2020).

Circular RNA could regulate gene expression at the transcriptional and post-transcriptional levels (Di Agostino et al., 2020; Dong et al., 2020; Jin et al., 2020; Li and Wang, 2020; Liu et al., 2020; Zhang et al., 2020a). CircAMOTL1 is found in human chromosome 11:94532555-94533477 and is upregulated in breast cancer tissues and could promote breast cancer progression (Ma et al., 2019; Ou et al., 2020). In cervical cancers, the role of circAMOTL1 is still little known, and we focused on the function and mechanism of circAMOTL1 in cervical cancer.

In this study, we found that circAMOTL1 was upregulated in cervical cancer tissues and cell lines. Overexpression of circAMOTL1 accelerated cervical cancer cell proliferation and migration, and restrained apoptosis, etc., while knockdown of circAMOTL1 restrained cervical cancer cell proliferation, migration, and facilitated apoptosis. As a putative target of circAMOTL1, miR-526b was predicted by multiple bioinformatics software and verified by further luciferase experiment, etc. Mechanistically, upregulation of circAMOTL1 restrained the relative expression of miR-526b and subsequently accelerated the expression of salt-inducible kinase 2 (SIK2) at posttranscriptional level. Taken together, our study uncovered the role of circAMOTL1 as a miRNA sponge in cervical cancers, and suggested that circAMOTL1 may be a potential therapeutic target in cervical cancer.

## Materials and Methods

### Patient Specimens

Our study included cervical carcinoma patients who received tumorectomy. We froze the cervical carcinoma specimens and paired normal peritumoral specimens in liquid nitrogen quickly after the resection. All the samples were obtained with the patients informed consents. No smoking subjects were included in our study. All the patients were diagnosed by at least two experienced pathologists. The non-tumorous tissue samples were at least 2 cm from the edge of the tumor, contained no obvious tumor cells, and were also evaluated by the pathologists. Anhui No. 2 Provincial People’s Hospital research ethics committee approved the experiment.

### Cell Lines and Cell Culture

Cervical carcinomas Hela and SiHa were obtained from the Institute of Cell Biology, Chinese Academy of Sciences in Shanghai. HcerEpic was purchased from YuChicell (Shanghai) Biological Technology Co., Ltd. Hela, SiHa, and HcerEpic cells were cultivated in DMEM (Gibco, United States). 1% antibiotics (100 U/ml penicillin and 100 μg/ml streptomycin sulfates) and 10% FBS were blended in DMEM and RPIM 1640. The atmosphere of the incubator is at 37°C and 5% CO_2_.

### Cell Transfection

Specific siRNA oligonucleotides were transiently transfected in Hela, SiHa, and HcerEpic cells, si-circAMOTL1 sense (5-CCGCGGTAACGAGTTGAAGATCCTCCTCGAGGAGGATCT TCAACTCGTTACCTTTTTG-3), si-circAMOTL1#2 sense (5-CCGCGTATGGGGTAACGAGTTGAAGCTCGAGCTTCAACT CGTTACCCCATACTTTTTG-3), si-SIK2 sense (forward 5-5-AGACCACCCTCACATAATCAAAC-3, reverse (5-AGACCAC CCTCACATAATCAAAC-3), si-NC, and si-RNA (si-circAMOTL1, si-SIK2) were obtained from Gene Pharma (Suzhou, China). The miR-526b mimic and inhibitor were synthesized by RiboBio Biotech (Guangzhou, China). Optimum density cervical carcinomas cells were cultivated and then transfected in six-well plates. The plasmid vectors (circAMOTL1, negative control) were obtained from Fubio Biological Technology Co. (Shanghai, China). Cells were transiently transfected using Lipofectamine3000 Transfection Reagent (Thermo Fisher, United States) based on the product descriptions. Cells were cultured for 24 h before transfection. The cells were then transiently transfected with the corresponding vectors using Lipofectamine 3000 Transfection Reagent based on the product descriptions. After 48 h, cells transfected with the corresponding vector were collected, such as for RT-qPCR.

### RT-qPCR

The total RNA was extracted from the specimens and cervical cells using TRIzol reagent (Invitrogen, United States). The cDNA was synthesized from whole RNA using the Prime Script RT Reagent Kit with gDNA Eraser (Takara, Dalian). SYBR Premix Ex Taq II (Takara, Dalian) was used to detect the expression levels of circAMOTL1 by RT-qPCR on the CFX96 sequence detection system (Bio-Rad). Supplementary Table S1 shows primer sequences. The endogenous controls were glyceraldehyde 3-phosphate dehydrogenase (GAPDH) and U6 small nuclear RNA. The relative quantification method (2^–ΔΔCt^) was used to calculate the expressions that have been normalized to endogenous controls.

### Cell Proliferation Assays

Cell proliferation was detected by CCK-8 (Beyotime, Shanghai), cells were incubated in a 96-well plate for 24 h, and then, respectively, transfected with siRNAs or plasmids in the CCK-8 assays. 0, 24, 48, and 72 h after transfection, the absorbance in each well was measured at by a microplate reader (Bio-Rad, United States).

### EdU Incorporation Assay

EdU Apollo DNA *in vitro* kit (RIBOBIO, Guangzhou) was also utilized cell proliferation that was by EdU incorporation assays. In a word, cells transfected siRNA or plasmid were hatched for 2 h at 37°C, and were hatched, respectively, with 100 μl of 50 μM EdU per well. In the end, fluorescence microscopy was applied to visualize the cells.

### Cell Migration Assay

The cells were implanted into the six-well plates and cultivated in the incubator. 100% confluence was obtained before the transfection with siRNA or plasma transfected cells. Use the sterilization 200 μl pet tips to generate clean lines in six-well plates. Use digital camera system to take photos in each well quickly. A day later, the picture was taken again. The travel distance was set at 0 and 24 h. Except for the difference of processing factor, it is synchronous, and it is carried out under the same conditions. The migration experiment calculates the relative migration distance.

### Flow Cytometry Assay

SiRNAs or plasmid vectors were, respectively, transfected in cervical carcinomas cells. 48 h after transfection, cells were collected and resuspended in fixation fluid 5 μl Annexin V-FIFC and 10 μl propidium iodide were added to 195 μl cell suspension. Flow cytometry (Beckman, United States) was used to detect cell apoptosis.

### Western Blot Analysis

Total proteins were separated by 10% SDS–PAGE and transferred to PVDF membranes. After blocking in the 5% non-fat milk and incubated overnight for 16 h in 4°C with the primary antibody. At room temperature for 2 h, the membranes were then incubated with a secondary antibody and enhanced chemiluminescence ECL kit (Beyotime, China) was visualized. β-actin, Tublin, or GAPDH was the internal standard. The details of antibodies are described in Supplementary Table S2.

### Dual-Luciferase Assays

Dual-Luciferase Reporter Assay System (Promega, United States) was used for the Dual-luciferase reporter assays. PmirGLO Dual-luciferase vectors, respectively, cloned the binding and mutant sequences (Fubio Biological Technology Co., Shanghai). CircAMOTL1 or SIK2 WT or Mut constructed and co-transfected along with miR-526b mimics or NC, then transfected with Lipofectamine 3000 and incubated for 48 h. Microplate reader was applied to measure the luciferase activities.

### Mouse Model Experiments

Our experiments were approved by the Institutional Ethics Review Board. Female BALB/c nude mice (5-week old) were divided into two groups and each group included five mice. LV-circAMOTL1 and LV-NC were purchased from Genechem (Shanghai, China). 4 × 10^6^ cells were injected into the mice dorsal flank regions. Every 5 days, tumor growth was measured. The formula, a^∗^b^2^/2 (a: long diameter, b: short diameter), was used to calculate tumor volume. In the end, we executed mice and observed the subcutaneous weight of each tumor.

### Transwell Migration and Invasion Assay

Cell invasion or migration rate was determined by transwell chamber with or without matrigel matrix (Corning, United States). The lower chamber was added with RPMI-1640 medium containing 20% FBS, while the transfected Hela and SiHa cells were injected into the upper one with 200 μl of serum-free medium and the whole steps were carried out according to the experiment instructor. In the end, paraformaldehyde was used to attach cells located on lower surface of the upper chamber and cells were analyzed under a microscope before stain with crystal violet.

### RNA Immunoprecipitation (RIP)

Magna RNA immunoprecipitation (RIP) RNA-Binding Protein Immunoprecipitation Kit (Millipore, United States) was used to detect the interaction between circAMOTL1 and miR-526b or miR-526b and SIK2. Hela and SiHa cells were collected and lyzed with RIP lysis buffer. Cell lysate was then incubated with anti-Argonaute2 (anti-Ago2) or normal rabbit IgG as the immunoprecipitating antibody overnight at 4°C. Purified RNA was analyzed by qRT-PCR. IgG was used as negative control.

### RNase R Treatment Assay

2 μg RNA and 6-unit RNase R (Geneseed Biotech, Guangzhou, China) were mixed and incubated at 37°C for 20 min. qRT-PCR for determining the mRNA level of circAMOTL1 and linear AMOTL1 before and after the RNase R treatment.

### Statistical Analysis

Every experimental assay was executed in triplicate. Triplicate biological replicates’ or samples’ data were presented as mean ± standard deviation (SD). SPSS 20.0 software (IBM, Chicago, IL, United States) was used to analyze assays’ statistical analyses. Paired samples *t*-test was used to analyze the circAMOTL1 and miR-526b expression. ANOVA was used to analyze CCK-8 assay data. The independent samples *t*-test was used to analyze other data. *P* < 0.05 was regarded as the statistically significant one.

## Results

### Higher Expression of CircAMOTL1 and Downregulated Expression of miR-526b in Cervical Carcinomas

**FIGURE 1:**
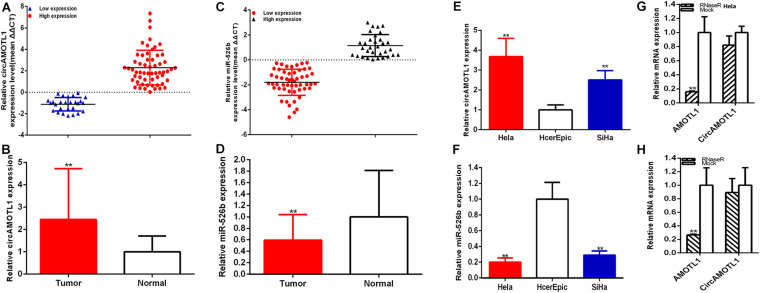
The relative expression of circAMOTL1 and miR-526b was detected in cervical carcinoma tissues and cells. The relative circAMOTL1 **(A,B)** and miR-526b **(C,D)** expression patterns were shown in paired cervical carcinomas tissues and normal tissues. The expression level in cervical carcinomas cells (Hela and SiHa) and HcerEpic **(E,F)** were detected. The expression level of circAMOTL1 and AMOTL1 mRNA treated with or without Rnase R was measured by qRT-PCR **(G,H)**. **P < 0.01.

The relative expression levels of circAMOTL1 and miR-526b were measured through RT-qPCR in cervical carcinoma samples. Compared with para-carcinoma specimens, the relative expression of circAMOTL1 was significantly increased about 2.446 times (58 of 85) of carcinoma samples (*P* < 0.001) (Figures 1A,B), and the relative expression of miR-526b was obviously decreased about 40.594% (53 of 85) of carcinoma samples (*P* < 0.001) (Figures 1C,D). Compared with HcerEpic cell, the relative expression of circAMOTL1 was higher expression in both cervical carcinoma cells, Hela about 3.680 times (*P* = 0.0081) and SiHa about 2.505 times (*P* = 0.0078) (Figure 1E), and the relative expression of miR-526b was obviously decreased in both cervical carcinoma cells, Hela about 80.032% (*P* = 0.0032) and SiHa about 71.166% (*P* = 0.0050) (Figure 1F). Concordantly, results of RNase R exonuclease treatment certified the circular nature of the circAMOTL1 (Figures 1G,H). Upregulated circAMOTL1 was closely associated with tumor differentiation (*P* = 0.019), FIGO staging (*P* = 0.002), and HPV16/18 (*P* = 0.015) in cervical carcinomas (Table 1). But age, tumor size, and lymph node metastasis were no obvious correlation with the expression level of circAMOTL1 and miR-526b. CircAMOTL1 could act as oncogene and miR-526b could act as the antioncogene in cervical carcinomas.

**TABLE 1:** Correlation between circAMOTL1 expression and clinicopathological characteristics of cervical cancer patients.

**Characteristics**	**Total**	**Expression of circAMOTL1**		***P*-value**
		**High**	**Low**	
		**(*n* = 58)**	**(*n* = 27)**	
**Tumor size (cm)**				
<4 cm	40	26 (65.0%)	14 (35.0%)	0.643
≥4 cm	45	32 (71.1%)	13 (28.9%)	
**Age**				
<60	32	21 (65.6%)	11 (34.4%)	0.811
≥60	53	37 (69.8%)	16 (30.2%)	
**Tumor differentiation**				
Medium-low	37	20 (54.1%)	17 (45.9%)	0.019*
High	48	38 (79.2%)	10 (20.8%)	
**FIGO staging**				
I	42	22 (52.4%)	20 (47.6%)	0.002**
II–IV	43	36 (83.7%)	7(16.3%)	
**HPV16/18**				
YES	70	52 (74.3%)	18 (25.7%)	0.015*
NO	15	6 (40.0%)	9 (60.0%)	
**Lymph node metastasis (N)**				
N0	75	50 (66.7%)	25 (33.3%)	0.492
N1 or above	10	8 (80.0%)	2 (20.0%)	

SCCA, squamous cell carcinoma antigen; FIGO staging, the Cervical Cancer Staging System of International Federation of Gynecology and Obstetrics in 2014. *P < 0.05. **P < 0.01.

### Knockdown of CircAMOTL1 Restrained Cervical Cell Lines Proliferation. Overexpression CircAMOTL1 Was on the Contrary

**FIGURE 2:**
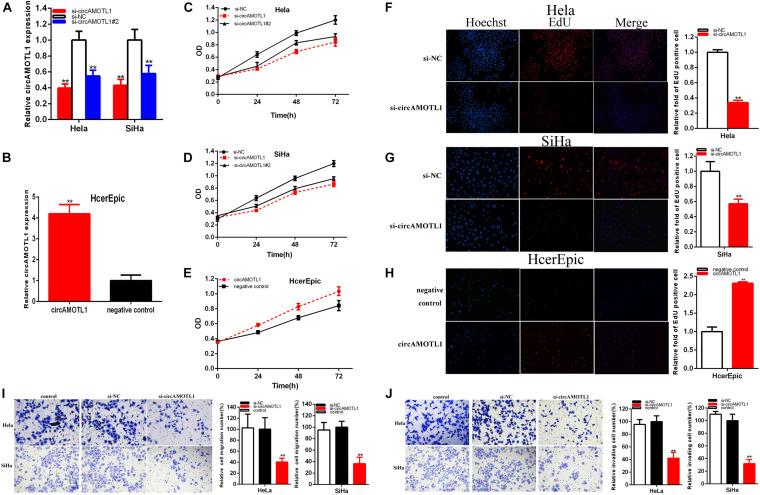
CircAMOTL1 acted as an oncogene. The relative expression level of circAMOTL1 was obviously decreased by si-circAMOTL1 and si-circAMOTL1#2 **(A)** and upregulated by circAMOTL1 **(B)**. Cell proliferation was detected in both cervical carcinomas cells after siRNA transfection **(C,D)** and circAMOTL1 **(E)**. Representative images of EdU assay and the relative fold changes of EdU positive cells were detected by siRNA **(F,G)** and circAMOTL1 **(H)**. Transwell assays showed that the relative cell migration and invasion was restrained after siRNA transfection in the Hela and SiHa **(I,J)** cell lines. **P < 0.01.

48 h after transfection of siRNA, miRNA mimics or inhibitor, and circAMOTL1, the relative expressions of circAMOTL1 and miR-526b were detected by qRT-PCR in Hela, SiHa, and HcerEpic. After transfection, the relative expression of circAMOTL1 was downregulated about 60.403% in Hela (*p* = 0.0010) and about 42.858% in SiHa (*P* = 0.0062) cells by si-circAMOTL1, and about 45.24% in Hela (*p* = 0.0041) and about 42.02% in SiHa (*P* = 0.0124) cells by si-circAMOTL1#2 (Figure 2A). And the relative expression of circAMOTL1 was notably increased about 4.1986 times in HcerEpic (*p* = 0.0004) after transfection of circAMOTL1 (Figure 2B).

Our study demonstrated that si-circAMOTL1 and si-circAMOTL1#2 (Figures 2C,D) obviously restrained both cervical carcinoma cells proliferation (*p* < 0.01). CircAMOTL1 (Figure 2E) remarkably facilitated HcerEpic cell proliferation (*p* < 0.01). Because the si-circAMOTL1 is more effective than si-circAMOTL1#2, we choose si-circAMOTL1 for the subsequent experiments.

EdU was elucidated cell proliferation. Compared with control group, EdU positive Hela and SiHa cells in si-circAMOTL1 group were reduced and circAMOTL1 group were reversed after transfection in HcerEpic cell line.

EdU assay proved that the EdU positive cells quantity was decreased about 66.078% in Hela (*P* < 0.001) (Figure 2F) and about 43.047% in SiHa (*P* < 0.001) (Figure 2G) in si-circAMOTL1 group. The quantity of EdU positive cells was increased about 2.300 times in HcerEpic (*P* < 0.001) (Figure 2H) of circAMOTL1 group.

Our study manifested that knockdown circAMOTL1 restrained cervical cell lines proliferation and overexpression circAMOTL1 facilitated cervical cell lines proliferation.

### Knockdown of CircAMOTL1 Restrained Cervical Cell Lines Migration. Overexpression CircAMOTL1 Facilitated Cervical Cell Line Migration

Cell migration was detected after transfection siRNA and plasmids by Transwell assay and scratch assay. Transwell assay indicated that the ratio of the relative migration was decreased by 59.62% in HeLa (*P* = 0.0092, si-circAMOTL1), and decreased by 63.73% in SiHa (*P* = 0.0019, si-circAMOTL1) (Figure 2I), and the ratio of the relative invasion were decreased by 57.75% in HeLa (*P* = 0.0015, si-circAMOTL1), and decreased by 68.10% in SiHa (*P* = 0.0006, si-circAMOTL1) (Figure 2J).

**FIGURE 3:**
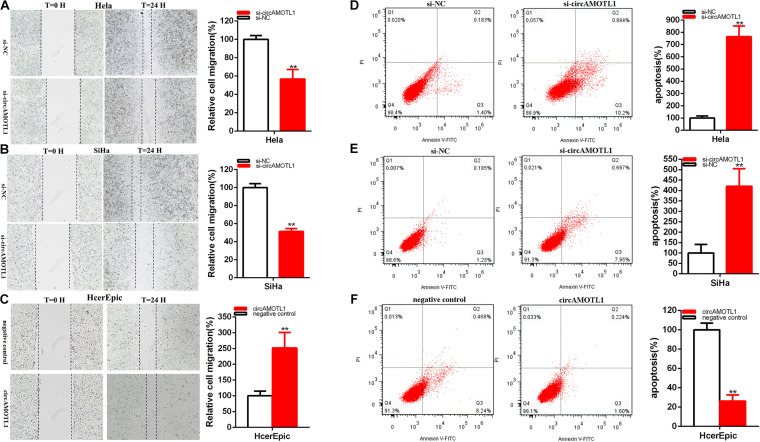
CircAMOTL1 acted as an oncogene. The relative cell migration was restrained after siRNA transfection in the Hela and SiHa **(A,B)** cell lines. The relative cell migration was facilitated after circAMOTL1transfection in the HcerEpic cell lines **(C)**. Apoptotic cells by flow cytometry analysis were measured after siRNA transfection in the Hela **(D)** and SiHa **(E)** cell lines, circAMOTL1 in the HcerEpic cell line **(F)**. **P < 0.01.

Scratch assay manifested that the ratio of the relative migration was decreased about 43.324% in Hela (*P* = 0.0026) (Figure 3A) and 48.65% in SiHa (*P* < 0.001) (Figure 3B) in si-circAMOTL1 group. The ratio of the relative circAMOTL1 group migration was upregulated about 2.516 times in HcerEpic (*P* = 0.0069) (Figure 3C).

Our results elucidated that knockdown of circAMOTL1 restrained cervical cell lines migration and invasion. Overexpression circAMOTL1 expression facilitated cervical cell lines migration.

### Knockdown of CircAMOTL1 Facilitated Cervical Cell Lines Apoptosis. Overexpression CircAMOTL1 Restrained Cervical Cell Lines Apoptosis

Flow cytometry assays were measured apoptosis. Compared with control groups, the apoptosis ratios were obviously upregulated about 7.647 times in Hela (*P* = 0.0002) and 4.203 times in SiHa (*P* = 0.0042) (Figures 3D,E) after transfection si-circAMOTL1.

Compared with control groups, the relative ratios of apoptosis were dramatically reduced about 74.41% in HcerEpic (*P* = 0.0018) (Figure 3F) after transfection circAMOTL1.

In brief, knockdown of circAMOTL1 facilitated cervical cell lines apoptosis and overexpression of circAMOTL1 restrained cervical cell lines apoptosis.

### MiR-526b Acts as the Tumor Suppressor Gene

**FIGURE 4:**
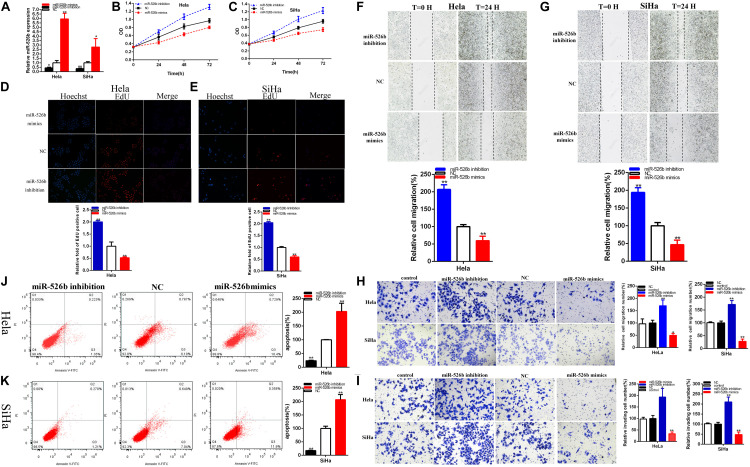
The relative expression level of miR-526b was significantly downregulated by miR-526b inhibitor and increased by miR-526b mimics **(A)**. Cell proliferation was detected after miR-526b inhibitor and miR-526b mimics transfection **(B,C)** in both cervical carcinoma cells. The relative EdU positive cells fold changes were detected by miR-526b inhibitor and miR-526b mimics **(D,E)**. The relative cell migration was restrained or accelerated after miR-526 mimics or inhibitor transfection in the Hela and SiHa **(F,G)** cell lines. The relative cell migration and invasion was detected after miR-526 mimics or inhibitor transfection in the Hela and SiHa **(H,I)** cell lines. Apoptotic cells were detected after miR-526 mimics or inhibitor transfection in the Hela and SiHa **(J,K)** cell lines. *P < 0.05, **P < 0.01.

The relative expression levels of miR-526b were remarkably reduced about 56.361% in Hela (*p* = 0.0240) and about 65.512% in SiHa (*p* = 0.0017) after transfection miR-526b inhibitor (Figure 4A). And the relative expression levels of miR-526b were obviously increased in about 5.961 times in Hela (*p* = 0.0002) and 2.781 times in SiHa (*p* = 0.0332) at after transfection miR-526b mimics (Figure 4A).

MiR-526b mimics notably restrained both cervical carcinoma cells proliferation (Figures 4B,C) (*p* < 0.01). MiR-526b inhibitor obviously accelerated both cervical carcinoma cells proliferation (Figures 4B,C) (*p* < 0.01).

The relative quantity of Edu positive cells in miR-526b mimics group was notably decreased about 47.581% in Hela (*P* = 0.0097) and 40.093% in SiHa (*P* = 0.0005) (Figures 4D,E). The relative quantity of Edu positive cells in miR-526b inhibitor group was upregulated about 2.003 times in Hela (*P* = 0.0007) and 2.0483 times in SiHa (*P* < 0.001) (Figures 4D,E). MiR-526b mimics restrained cervical carcinomas cells proliferation and miR-526b inhibition accelerated cervical carcinoma cells proliferation.

The ratio of the relative migration was downregulated about 40.308% in Hela (*P* = 0.0077) and 53.40% in SiHa (*P* = 0.0041) (Figures 4F,G) after transfection miR-526b mimics. The ratio of the relative migration was upregulated about 2.068 times in Hela (*P* = 0.0002) and 1.939 times in SiHa (*P* = 0.0006) (Figures 4F,G) after transfection miR-526b inhibitor. Transwell assays elucidated that the ratio of the relative migration was decreased by 50.33% in HeLa (*P* = 0.0032, miR-526b mimics), and decreased by 72.19% in SiHa (*P* = 0.0004, miR-526b mimics) (Figure 4H). The ratio of the relative migration was upregulated about 1.701 times in Hela (*P* = 0.0097, miR-526b inhibition) and 1.715 times in SiHa (*P* = 0.0019, miR-526b inhibition) (Figure 4H). The ratio of the relative invasion was decreased by 66.20% in HeLa (*P* = 0.0017, miR-526b mimics) and 51.93% in SiHa (*P* = 0.0048, miR-526b mimics) (Figure 4I). The ratio of the relative invasion was upregulated about 1.933 times in Hela (*P* = 0.0148, miR-526b inhibition) and 2.103 times in SiHa (*P* = 0.0019, miR-526b inhibition) (Figure 4I).

Upregulated miR-526b expression suppressed cervical carcinoma cells migration and invasion, and depression miR-526b accelerated cervical carcinomas cells migration and invasion.

Compared with NC groups, the ratios of the relative apoptosis were obviously went up about 2.208 times in Hela (*P* = 0.0041) and 2.069 times in SiHa (*P* = 0.0009) (Figures 4J,K) after transfection miR-526b mimics. Compared with NC groups, the ratios of the relative apoptosis were obviously lessened about 76.10% in Hela (*P* < 0.0001) and 82.34% in SiHa (*P* < 0.0001) (Figures 4J,K) after transfection miR-526b inhibitor. Ultimately, upregulated miR-526b expression accelerated cervical carcinomas cells apoptosis and reduction miR-526b restrained cervical carcinomas cells apoptosis.

### CircAMOTL1 Sponges miR-526b

**FIGURE 5:**
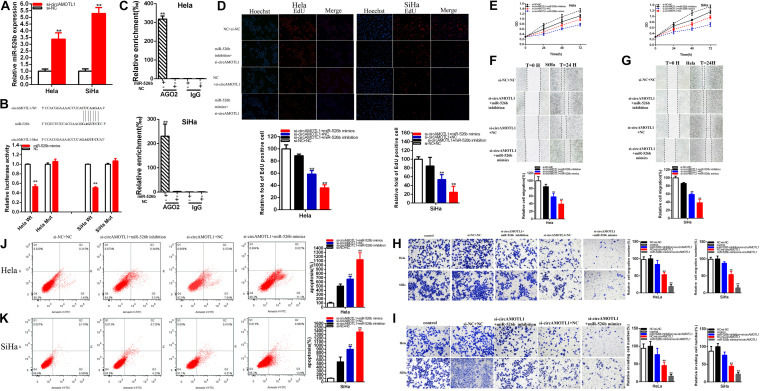
CircAMOTL1 was a target of miR-526b. The relative expression of miR-526b was increased by si-circAMOTL1 **(A)**. Dual-luciferase reporter assays were detected in Hela and SiHa cells co-transfected with circAMOTL1-Wt or circAMOTL1-Mut and miR-526b mimics or NC **(B)**. Ago2-RIP assay demonstrated the enrichment of circAMOTL1, and miR-526b in anti-Ago2 group compared to the negative control anti-IgG **(C)**. Cell proliferation was detected after co-transfection with si-NC + NC in both cervical carcinoma cell lines, si-circAMOTL1 + miR-526b inhibitor, or si-circAMOTL1 + miR-526b mimics by Edu and CCK-8 **(D,E)**. The relative cell migration after co-transfection with si-NC + NC, si-circAMOTL1 + miR-526b inhibitor, or si-circAMOTL1 + miR-526b mimics was as follows **(F,G)**. The relative cell migration and invasion was detected after co-transfection with si-C + NC, si-circAMOTL1 + miR-526b inhibitor, or si-circAMOTL1 + miR-526b mimics in the Hela and SiHa **(H,I)** cell lines. The apoptotic cells were measured after co-transfection with si-C + NC, si-circAMOTL1 + miR-526b inhibitor, or si-circAMOTL1 + miR-526b mimics **(J,K)**. **P < 0.01.

Compared with si-NC groups, the relative expression of miR-526b was increased about 3.380 times in Hela (*P* = 0.0012) and 5.293 times in SiHa (*P* < 0.001) (Figure 5A) in si-circAMOTL1 groups. Bioinformatics databases were used to predict some underlying binding sites of circAMOTL1 with miR-526b. The predictions were verified by luciferase reporter assay. MiR-526b mimics obviously suppressed circAMOTL1 wild type reporter luciferase activity; compared with the co-transfections NC + pmirGLO-circAMOTL1-Wt, the luciferase activity was obviously reduced about 46.850% in Hela (*P* < 0.001) and 45.88% in SiHa (*P* = 49.112) in the co-transfections miR-526b mimics + pmirGLO-circAMOTL1-Wt; nevertheless, miR-526b could not suppress the circAMOTL1mutant reporter vector luciferase activity (Figure 5B). The luciferase reporter assays were verified that circAMOTL1 sponged miR-526b. CircAMOTL1 and miR-526b were enriched by antibody against Ago2 in contrast with immunoglobulin G (IgG) antibody (Figure 5C).

### CircAMOTL1 Sponging miR-526b Mediated Cervical Carcinoma Cell Progression

That si-circAMOTL1 co-transfected miR-526b mimics could manifest more powerful suppressed effects on cervical carcinoma cells proliferation (Figures 5D,E) and migration (Figures 5F,G) than si-NC co-transfection with NC (si-NC + NC); meanwhile, compared with si-NC + NC group, apoptosis was obviously accelerated in si-circAMOTL1 co-transfection miR-526b mimics(si-circAMOTL1 + miR-526b) group (Figures 5J,K). Conversely, miR-526b inhibitor could partially reverse inhibited effects on cervical carcinoma cells progression induced by si-circAMOTL1.

Compared with si-NC + NC, si-circAMOTL1 co-transfected miR-526b mimics could obviously decrease the relative quantity of Edu positive cells about 64.080% in Hela (*P* = 0.0002) and 75.610% in SiHa (*P* = 0.0009). Moreover, miR-526b inhibitor could partially reverse inhibited effects on cervical carcinoma cells proliferation induced by si-circAMOTL1 and increased about 22.702% in Hela and 29.277% in SiHa cell lines (Figure 5D).

The CCK-8 assays have been manifested that si-circAMOTL1 co-transfected miR-526b mimics remarkably restrained both cervical carcinoma cells proliferation in (*p* < 0.01 in Hela and SiHa). Meantime, miR-526b inhibitor could partially reverse inhibited effects on cervical carcinoma cells proliferation induced by si-circAMOTL1 (Figure 5E).

Compared with si-NC + NC, si-circAMOTL1 co-transfected miR-526b mimics could obviously decrease the ratio of the relative migration about 65.487% in Hela (*P* = 0.0017) and 61.355% in SiHa (*P* = 0.0003). Moreover, miR-526b inhibitor could partially reverse inhibited effects on cervical carcinoma cells migration induced by si-circAMOTL1 and increased about 23.673% in Hela and 20.518% in SiHa cell lines (Figures 5F,G).

Transwell assays revealed that, compared with si-NC + NC, si-circAMOTL1 co-transfected miR-526b mimics could obviously decrease the ratio of the relative migration about 81.10% in Hela (*P* = 0.0002) and 85.06% in SiHa (*P* = 0.001). Furthermore, miR-526b inhibitor could partially reverse inhibited effects on cervical carcinoma cells migration induced by si-circAMOTL1 (Figure 5H).

Transwell assays elucidated that, compared with si-NC + NC, si-circAMOTL1 co-transfected miR-526b mimics could obviously decrease the ratio of the relative invasion about 84.35% in Hela (*P* = 0.0005) and 77.88% in SiHa (*P* = 0.0002). Moreover, miR-526b inhibitor could partially reverse inhibited effects on cervical carcinoma cells invasion induced by si-circAMOTL1 (Figure 5I).

Compared with si-NC + NC, si-circAMOTL1 co-transfected miR-526b mimics could obviously accelerate the ratio of the relative apoptosis about 13.34 times in Hela (*P* = 0.0004) and 14.2 times in SiHa (*P* < 0.001). Furthermore, miR-526b inhibitor could partially reverse promoting apoptosis on cervical carcinoma cells migration induced by si-circAMOTL1 and reduced about 164.69% in Hela and 342.99% in SiHa (Figures 5J,K).

### CircAMOTL1 Sponging miR-526b Closely Regulated SIK2 and EMT

**FIGURE 6:**
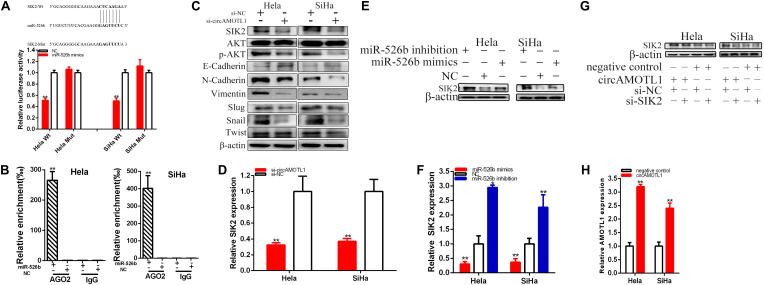
CircAMOTL1 positively regulates SIK2 expression via sponging miR-526b. SIK2-Wt and miR-526b mimics co-transfection obviously restrained luciferase activity **(A)** by dual-luciferase reporter assay. Ago2-RIP assay demonstrated the enrichment of SIK2 and miR-526b in anti-Ago2 group compared to the negative control anti-IgG **(B)**. Knockdown of circAMOTL1 downregulated SIK2 expression in cervical carcinomas cells **(C)**. The relative expression level of SIK2 was obviously decreased by si-circAMOTL1 **(D)**. Overexpression miR-526b downregulated SIK2 expression and knockdown of miR-526b upregulated SIK2 expression in cervical carcinoma cells **(E)**. The relative expression level of SIK2 was obviously decreased by miR-526b mimics and increased by miR-526b inhibition **(F)**. Si-SIK2 obviously reversed circAMOTL1 expression inhibition induced by overexpression circAMOTL1 in cervical carcinoma cells **(G)**. The relative expression of AMOTL1 was detected by overexpression circAMOTL1 in cervical carcinoma **(H)**. **P < 0.01.

Bioinformatics databases were used to predict SIK2 with miR-526b possible mutual binding sites (Figure 6A). The predicted binding sites and binding effects were manifested through luciferase reporter assay.

Compared with the co-transfections with NC + pmirGLO-circAMOTL1-Wt, our results have been confirmed that miR-526b mimics dramatically restrained SIK2 wild type reporter luciferase activity, which lessened about 48.804% in Hela (*p* = 0.0001) and 49.881% in SiHa (*p* = 0.0003) in the co-transfection with miR-526b mimics + SIK2C-3′UTR-Wt. Inversely, miR-526b could not restrain the SIK2 mutant binding sites reporter vector luciferase activity (Figure 6A).

SIK2 and miR-526b were enriched by antibody against Ago2 in contrast with IgG antibody (Figure 6B).

Our study manifested that the circAMOTL1 expression was closely related to SIK2 expression and downregulated circAMOTL1 could reduce SIK2 expression in cervical carcinomas cells. Our further experiments confirmed that restrained circAMOTL1 could refer to AKT signaling in cervical carcinomas cells (Figure 6C). Knockdown of circAMOTL1 decreased SIK2, p-AKT, N-Cadherin, Vimentin, Slug, Snail, Twist, and upregulated E-Cadherin expression in cervical carcinoma cells. Compared with si-NC groups, the SIK2 expressions were decreased about 67.57% in Hela (*P* = 0.0039) and 63.08% in SiHa (*P* = 0.0022) (Figure 6D) in si-circAMOTL1 groups. Overexpression miR-526b reduced SIK2 expression and miR-526b inhibition upregulated SIK2 expression in cervical carcinoma cells (Figure 6E). CircAMOTL1 could closely regulate SIK2 expression via sponging miR-526b in cervical carcinoma cells. The relative expression levels of SIK2 were remarkably reduced about 69.40% in Hela (*p* = 0.0145) and 63.44% in SiHa (*P* = 0.0086) after transfection miR-526b mimics (Figure 6F). And the relative expression levels of SIK2 were obviously increased in about 2.949 times in Hela (*p* = 0.0003) and 2.262 times in SiHa (*p* = 0.0097) after transfection miR-526b inhibition (Figure 6F). Further experiments have been manifested that knockdown of SIK2 dramatically reversed the promotion of SIK2 expression induced by overexpressing circAMOTL1 in cervical carcinoma cells (Figure 6G). AMOTL1 expression was upregulated by overexpressing circAMOTL1 in cervical carcinoma cells (Figure 6H).

### Silencing of SIK2 Reverses Malignant Cervical Carcinomas Cells Phenotypes Promotion of Overexpression CircAMOTL1

**FIGURE 7:**
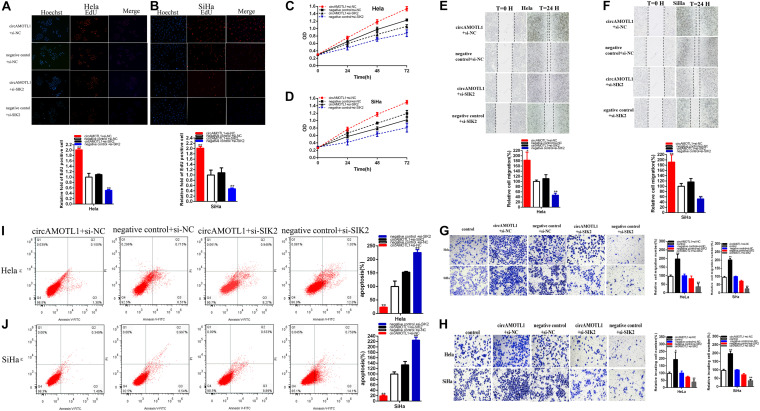
CircAMOTL1 positively regulates SIK2 expression via sponging miR-526b. Knockdown SIK2 significantly reversed cell proliferation promotion induced by overexpression circAMOTL1 (EdU, **A,B**, CCK8, **C,D**). Knockdown SIK2 significantly reversed cell migration promotion induced by overexpression circAMOTL1 **(E,F)**. Transwell assays showed that knockdown SIK2 significantly reversed cell migration and invasion promotion induced by overexpression circAMOTL1 **(G,H)**. Knockdown SIK2 obviously reversed cell apoptosis inhibition induced by overexpression circAMOTL1 **(I,J)**. *P < 0.05, **P < 0.01.

Moreover, knockdown of SIK2 obviously reversed cervical carcinoma cells proliferation promotion (Figures 7A–D) induced by overexpression circAMOTL1. And SIK2 knockdown could obviously reverse cervical carcinoma cells migration (Figures 7E,F) induced by overexpression circAMOTL1. And SIK2 knockdown could obviously reverse cervical carcinoma cells migration and invasion (Figures 7G,H) induced by overexpression circAMOTL1. Meanwhile, SIK2 knockdown could notably reverse cervical carcinoma cells apoptosis suppression (Figures 7I,J) induced by overexpression circAMOTL1. CircAMOTL1 could accelerate malignant cervical carcinoma cells phenotypes via SIK2-dependent manner.

### Knockdown of CircAMOTL1 Suppressed Cervical Carcinomas Cells Tumorigenicity

**FIGURE 8:**
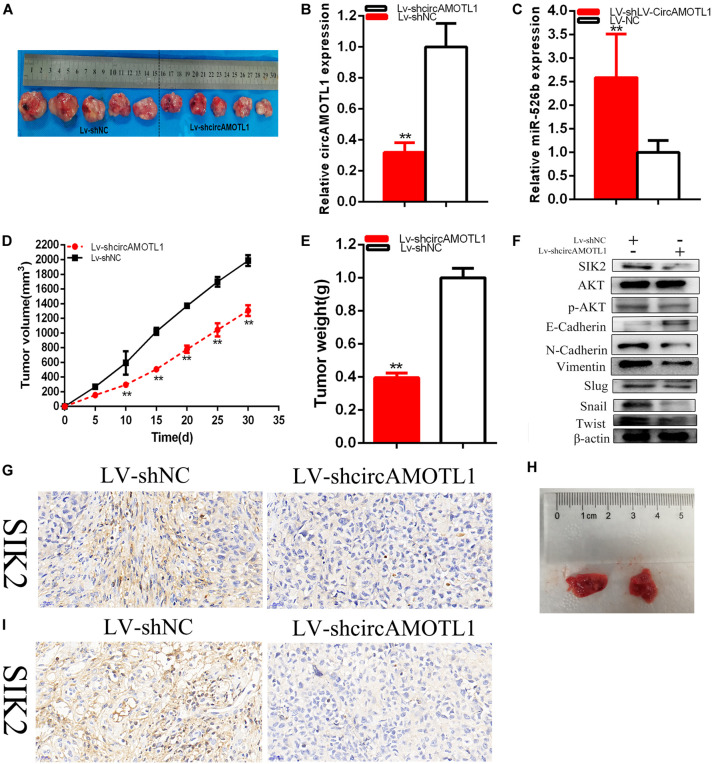
The circAMOTL1 effect on cervical carcinomas cells tumorigenicity. Tumors collected from mice were showed **(A)**. The relative expression level of circAMOTL1 was obviously decreased and miR-526b expression was upregulated by LV-circAMOTL1 **(B,C)**. Tumor volume curve was measured and analyzed **(D)**. Tumor weight was detected **(E)**. Knockdown of circAMOTL1 downregulated SIK2 **(F)**. Knockdown of circAMOTL1 downregulated SIK2 expression *in vivo* of cervical carcinoma cells **(G)**. The number and size of pulmonary metastases in the LV-circAMOTL1 group were significantly reduced compared with those in the LV-shNC group **(H)**. Knockdown of decreased SIK2 expression in pulmonary metastases **(I)**. **P < 0.01.

Generation of xenograft was used to confirm whether circAMOTL1 regulated tumorigenicity of cervical carcinoma cells. Knockdown of circAMOTL1 could restrain the cervical carcinoma cells tumorigenicity *in vivo* (Figures 8A–G). Solid tumors were obtained from mice were as shown as Figure 8A. The relative expression levels of circAMOTL1 (Figure 8B) and miR-526b (Figure 8C) were detected, circAMOTL1 was obviously reduced, and miR-526b was upregulated in LV-shcircAMOTL1 groups compared with LV-shNC group of cervical carcinoma cells *in vivo*. Tumor growth was slower in LV-shcircAMOTL1 groups than LV-shNC groups *in vivo* (Figure 8D). Tumor weight was decreased in LV-shcircAMOTL1 groups than LV-shNC groups *in vivo* (Figure 8E). Knockdown of circAMOTL1 could reduce SIK2, p-AKT, N-Cadherin, Vimentin, and upregulated E-Cadherin expression of cervical carcinoma cells *in vivo* (Figure 8F). Knockdown of circAMOTL1 restrained SIK2 expression by IHC experiments (Figure 8G) of cervical carcinoma cells *in vivo*. CircAMOTL1 facilitated cervical carcinoma cells tumorigenicity via upregulating SIK2 expression. Knockdown of circAMOTL1 significantly reduced the number and size of pulmonary metastases (Figure 8H). Moreover, we found that knockdown of circAMOTL1 inhibited SIK2 expression (Figure 8I). The results indicated that circAMOTL1 promotes cervical carcinoma metastasis *in vivo*.

**FIGURE 9:**
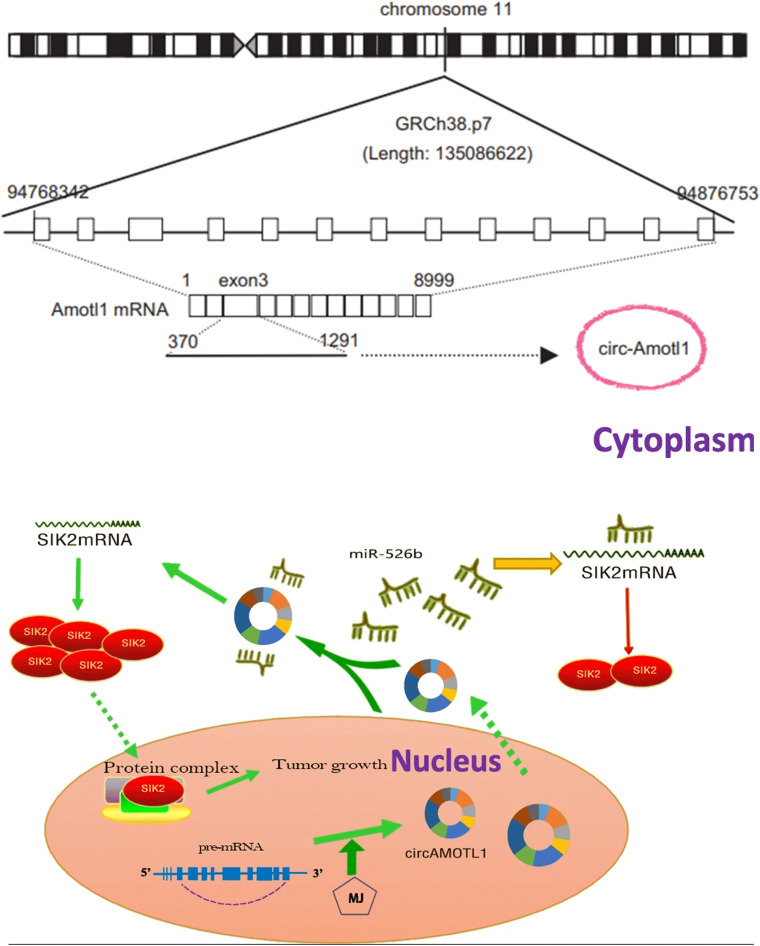
The schematic diagram of the oncogenic role of circAMOTL1 in cervical carcinomas cells. CircAMOTL1 promotes tumor progression in cervical cancers by sponging miR-526b to upregulate SIK2. CircAMOTL1 functions as an miRNA sponge to positively regulate SIK2 expression through sponging miR-526b and subsequently promotes malignant phenotypes of cervical carcinomas cells, thus playing an oncogenic role in cervical carcinoma pathogenesis.

As simulated diagram shown as Figure 9, circAMOTL1 was dramatically elevated in cervical carcinoma cells and circAMOTL1 could sponge miR-526b to closely regulate SIK2 expression. CircAMOTL1 promotes tumor progression in cervical cancer by sponging miR-526b to upregulate SIK2. Upregulated SIK2 protein could facilitate transcription and translation of proteins operating through indispensably abnormal protein signaling pathways, and subsequently could accelerate malignant cervical carcinoma cells phenotypes.

## Discussion

Non-coding RNAs have been manifested not to be the transcriptional noise, and but to be the important regulatory molecules (Zhuang et al., 2015; Chen et al., 2016, 2017; Li et al., 2016a,b; Tran et al., 2020), and circRNAs are novel endogenous non-coding RNAs that have been identified as owing key regulatory roles in the cancer biology (Ma et al., 2019; Di Agostino et al., 2020; Dong et al., 2020; Jin et al., 2020; Li and Wang, 2020; Liu et al., 2020; Ou et al., 2020; Zhang et al., 2020a). circRNAs act as important roles in the development and progression of cancers and are involved in various biological processes, such as cell proliferation, apoptosis, and migration by regulating gene expression (Liu et al., 2019; Di Agostino et al., 2020; Ji et al., 2020; Tang and Chen, 2020; Wang et al., 2020c). Mangy circRNAs have been reported to play important functions in cervical cancers, such as hsa_circ_0000515 (Tang and Chen, 2020), has_ circ_0000388 (Meng et al., 2020), circ-HIPK3 (Qian and Huang, 2020), circ-ITCH (Li et al., 2020; Wu et al., 2020b), circCLK3 (Jin et al., 2020), etc. However, the role and mechanism of circAMOTL1 in the cervical cancer are thoroughly unclear. As a 922nt RNA, circAMOTL1 is transcribed from a circular form of AMOTL1 in human chromosome 11. Some studies suggested that circAMOTL1 functioned as an oncogene in breast cancer and prostate cancer (Ma et al., 2019; Ou et al., 2020). However, the role of circAMOTL1 in cervical cancer remains completely unclear. Our study focused on circAMOTL1 in cervical cancer and found that circAMOTL1 was upregulated in cervical cancer tissues and cell lines. Overexpression of circAMOTL1 facilitated cervical cell proliferation, migration, and reduced apoptosis, and knockdown of circAMOTL1 restrained cervical cancer cell proliferation, migration, and promoted apoptosis. Mechanistically, through bioinformatics prediction software, we identified that miR-526b interacted with the target sites on circAMOTL1, and the interaction was detected by luciferase report assay.

Furthermore, the function of miR-526b in the development of cancers is already uncovered. In the oral squamous cell carcinoma (OSCC) (Zhao et al., 2020b) and hepatocellular carcinoma (HCC) (Wei et al., 2020), miR-526b could function as a tumor suppressor, inhibited cell growth by targeting c-Myc, and miR-526b also acts as a tumor suppressor in gastric cancer by targeting YAP1. Furthermore, miR-526b interacts with several ncRNA, such as hsa_circ_0091581 (Wei et al., 2020), circ_SPECC1 (Chen and Wang, 2019), lincRNA-NR_024015 (Han et al., 2017), etc. However, the relationship between miR-526b and circAMOTL1 in cervical cancers is totally unknown. Through bioinformatics analysis, we identified a putative binding site between circAMOTL1 and miR-526b, and find that circAMOTL1 acts as the sponge of miR-526b, which can directly bind to miR-526b and regulate its target genes of SIK2. Ulteriorly, circAMOTL1 overexpression could lead to miR-526b downregulation while circAMOTL1 knockdown upregulates the expression level of miR-526b. MiR-526b inhibitor could partially reverse effects and miR-526b mimics could enhance effects induced by knockdown of circAMOTL1 on cervical carcinoma cells.

In a word, we proved that miR-526b restrained the growth of cervical cancer cells and mediates the function of circAMOTL1 in cervical cancers. The overexpression of circAMOTL1 reduced the expression of miR-526b and subsequently accelerated the expression level of SIK2 at the post-transcriptional level. SIK2 belongs to the AMP-activated protein kinase family that regulates a variety of biological functions, including fatty acid oxidation. SIK2 has been established as a regulator of many biological processes, including cell metabolism. Some literatures manifested that SIK2 acted as the oncogene and the crucial regulator of glucose metabolism in ovarian cancer cells through PI3K/AKT/HIF-1α pathway, and SIK2 acted as a critical regulator of lipid synthesis and promotes ovarian cancer growth (Chen et al., 2019; Kim et al., 2019; Gao et al., 2020; Zhao et al., 2020a). In our research, we verified that miR-526b directly targets the SIK2 to reduce the protein levels of SIK2 in cervical cancer. And the protein level of SIK2 was upregulated upon circAMOTL1 overexpression and mediated the function of circAMOTL1. Silencing of SIK2 reversed malignant cervical carcinomas cells phenotypes promotion of overexpression circAMOTL1. Some studies illustrated that circRNAs and miRNAs could play important roles in cervical cancers (Jayamohan et al., 2019; Dong et al., 2020; Jin et al., 2020; Li et al., 2020; Ou et al., 2020; Tang and Chen, 2020; Tran et al., 2020; Zhang et al., 2020a). Previously, the function of circAMOTL1 in cervical cancers is completely unknown. We speculate that circAMOTL1 could sponge miR-526b as the ceRNA method. In conclusion, we have demonstrated that circAMOTL1 was upregulated in cervical cancer tissues and enhanced cervical cancer progression by promoting cell proliferation, migration, and reducing apoptosis. Mechanistically, overexpression of circAMOTL1 decreased the expression of miR-526b and subsequently promoted the expression of SIK2 and epithelial–mesenchymal transition (EMT) at posttranscriptional level. Taken together, our study reveals that circAMOTL1 acts as an oncogene by miRNA sponge in cervical cancers, and indicates that circAMOTL1 may be a potential therapeutic target in cervical cancers.

## Conclusion

The experiments have been manifested that circAMOTL1 could sponge miR-526b to closely regulate SIK2 expression and subsequently accelerate the malignant cervical carcinomas cells’ phenotypes and EMT, and therefore could act as a carcinogene in the cervical carcinoma mechanism. Our experiments could provide some useful directions to further explore its pathogenesis of the cervical carcinoma progression and development. In conclusion, the experiments manifested that circAMOTL1-miR-526b-SIK2 axis could play some significant roles in the progression and development of cervical carcinomas. CircAMOTL1 and miR-526b are novel and important tumor biomarkers, which could be some underlying diagnostic biomarkers and remedial targets for malignant cervical carcinomas in the future.

## Data Availability Statement

The original contributions presented in the study are included in the article/Supplementary Material. Further inquiries can be directed to the corresponding author.

## Ethics Statement

The studies involving human participants were reviewed and approved by Anhui No. 2 Provincial People’s Hospital. The patients/participants provided their written informed consent to participate in this study. The animal study was reviewed and approved by Anhui No. 2 Province People’s Hospital.

## Author Contributions

MC, ZS, and RM designed and performed the experiments, wrote and reviewed the manuscript. ZS, RM, SN, FX, and WZ involved to collect data. All authors listed have made a substantial, direct and intellectual contribution to the work, and approved it for publication and contributed to the article and approved the submitted version.

## Conflict of Interest

The authors declare that the research was conducted in the absence of any commercial or financial relationships that could be construed as a potential conflict of interest.
