# Supplementary material for: CircAMOTL1 Promotes Tumorigenesis Through miR-526b/SIK2 Axis in Cervical Cancer
Source: Front Cell Dev Biol. 2020 Dec 3;8:568190. doi: 10.3389/fcell.2020.568190 (PMC7744824; doi:10.3389/fcell.2020.568190)
Supplement: Supplementary file 1 [file Data_Sheet_1.docx]

**Electronic supplementary material (ESM) Table S**

ESM Table S1: The primers for real-time QPCR

| Gene | Forward or Reverse | Primer sequence |
| --- | --- | --- |

| circAMOTL1 | Forward | 5’- GATGGTCAAGCCCTACCCTG -3’ |
| --- | --- | --- |
|  | Reverse | 5’- -CCCTGATGCTACTGGTTGCC -3’ |
| miR-526b  SIK2  GAPDH | Forward  Reverse  Forward  Reverse  Forward | Provided by RiboBio  Provided by RiboBio  AGACCACCCTCACATAATCAAAC  ATTTTCGCCTGGCTTCAGACT  5’-CGCTCTCTGCTCCTCCTGTTC-3’ |
|  | Reverse | 5’ATCCGTTGACTCCGACCTTCAC-3’ |
| U6 | Forward | 5’-CTCGCTTCGGCAGCACA-3’ |
|  | Reverse | 5’- ACGCTTCACGAATTTGCGT-3’ |
| AMOTL1 | Forward | 5′-GTCTACCACCAAGCGAGAATCG-3′ |
|  | Reverse | 5′-CTGCTGGATAGTTGCCTGTTAGC-3′ |

Table S2: Antibodies used for Western blots

| Antibody | Description | Dilution | Supplier | Country |
| --- | --- | --- | --- | --- |
| Anti-SIK2 | Mouse monoclonal | 1:1000 | Santa Cruz | USA |
| Anti- AKT | Mouse monoclonal | 1:1000 | Santa Cruz | USA |
| Anti-p-AKT | Mouse monoclonal | 1:500 | Santa Cruz | USA |
| Anti-E-Cadherin  Anti-N-Cadherin  Anti-Vimentin  Anti-β-Actin  Anti-Twist  Anti-Slug  Anti-Snail | Mouse monoclonal  Mouse Monoclonal  Mouse Monoclonal  Mouse Monoclonal  Mouse monoclonal  Mouse monoclonal  Mouse monoclonal | 1: 500  1: 1000  1: 1000  1: 1000  1:500  1:500  1:500 | Santa Cruz  Santa Cruz  Santa Cruz  Beyotime  Santa Cruz  Santa Cruz  Santa Cruz | USA  USA  USA  China  USA  USA  USA |
